# Primary squamous cell carcinoma of the ampulla of Vater: a case report

**DOI:** 10.1186/s40792-016-0130-0

**Published:** 2016-01-08

**Authors:** B. Balci, B. Calik, T. Karadeniz, H. Sahin, L. Ugurlu, C. Aydin

**Affiliations:** Department of General Surgery, Izmir Tepecik Education and Research Hospital, Izmir, Turkey; Department of Pathology, Izmir Tepecik Education and Research Hospital, Izmir, Turkey; Department of Radiology, Izmir Tepecik Education and Research Hospital, Izmir, Turkey

**Keywords:** Ampulla of Vater, Squamous cell carcinoma

## Abstract

**Background:**

Primary squamous cell carcinoma of the ampulla of Vater is a very rare type of tumor, and the prognosis is not well known mainly due to a limited number of cases reported. Here, we aimed to report a case with primary squamous cell carcinoma of the ampulla of Vater.

**Case presentation:**

A 54-year-old woman presented with weight loss, jaundice, and pain in the epigastric and right upper quadrant of the abdomen. With extensive radiological imaging, the patient was diagnosed with periampullary tumor and Whipple’s procedure was performed. The immunohistochemical analyses supported the diagnosis of primary squamous cell carcinoma. The postoperative course was uneventful. The patient was discharged, and adjuvant chemotherapy was recommended.

**Conclusion:**

Primary squamous cell carcinoma of the ampulla of Vater is a very rare histological type with an unclear pathogenesis. A better understanding of pathogenesis might be helpful in optimizing the treatment for this specific rare type of tumor.

## Background

Periampullary cancers include a group of malignant tumors arising in the pancreas, the distal common bile duct, the ampulla of Vater, and the duodenum. Pathologic examination of resected pancreaticoduodenectomy specimens reveal that 40–60 % are adenocarcinomas of the head of the pancreas, 10–20 % are adenocarcinomas of the ampulla of Vater, 10 % are distal bile duct adenocarcinomas, and 5–10 % are duodenal adenocarcinomas [1].

The most common histopathology of tumors in the ampulla of Vater is adenocarcinomas followed by adenosquamous [2–4] and squamous cell carcinomas. To our knowledge, there are only four case reports with primary squamous cell carcinoma [5–8] and one case report with co-existent primary squamous cell carcinoma and adenocarcinoma in the ampulla of Vater [9]. Here, we aimed to report a case with primary squamous cell carcinoma of the ampulla of Vater.

## Case presentation

**Fig. 1:**
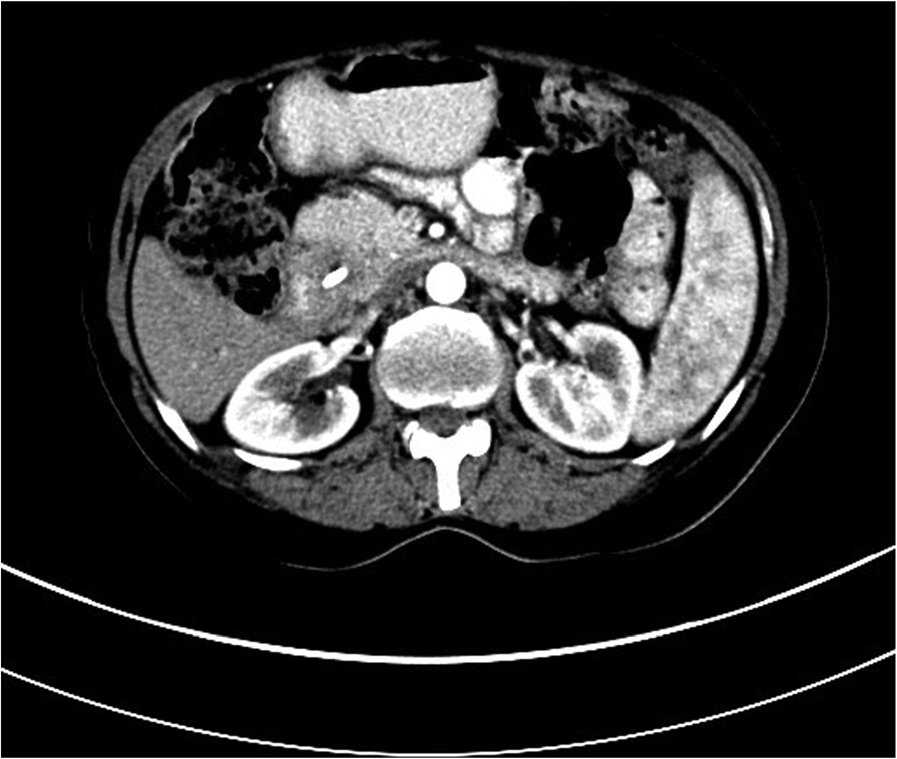
Arterial phase CT scan of a 54-year-old woman shows a high-density stent in the bile duct and a hypodense tumoral lesion in the periampullary region

A 54-year-old woman applied to an out medical center with the complaints of weight loss, jaundice, and pain in the epigastric and right upper quadrant of the abdomen. Computer tomography (CT) scan revealed a mass with a size of 13 mm in the ampullary region consistent with periampullary tumor (Fig. 1).

The patient underwent an endoscopic retrograde cholangiopancreatography (ERCP) procedure which revealed significant dilatation in the middle and distal segments of the common bile duct together with an abrupt ending in the distal segment of the common bile duct. A plastic stent was inserted to the common bile duct via ERCP, and multiple biopsies were taken from the periampullary region. The histopathological result was squamous cell carcinoma. The patient was referred to our hospital for further investigations.

The physical examination of the patient was unremarkable. Laboratory tests revealed elevated ALP (200 U/l; normal range, 30–120 U/l) and GGT (181 U/l; normal range, 0–38 U/l) levels. Billirubin level was within the normal limits. The serum level of the tumor markers of CEA and CA-125 were 2.41 and 14.23 ng/ml (normal range, 0–35 U/ml), respectively. CA-19-9 was 47.47 U/ml (normal range, 0–27 U/ml).

**Fig. 2:**
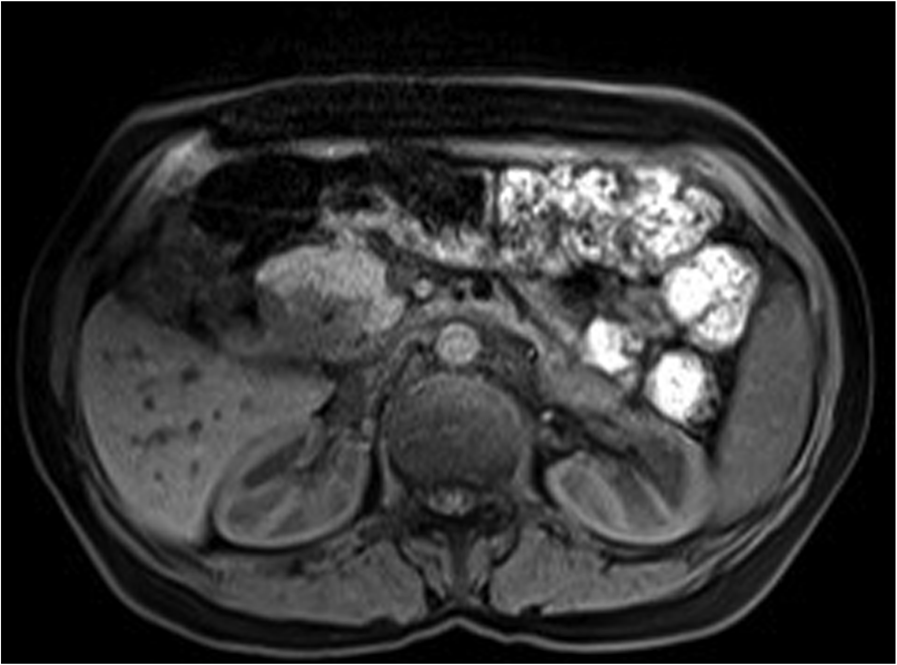
Contrast-enhanced T1-weighted MRI shows a hypointense lesion in the periampullary region near normal hyperintense pancreatic tissue

**Fig. 3:**
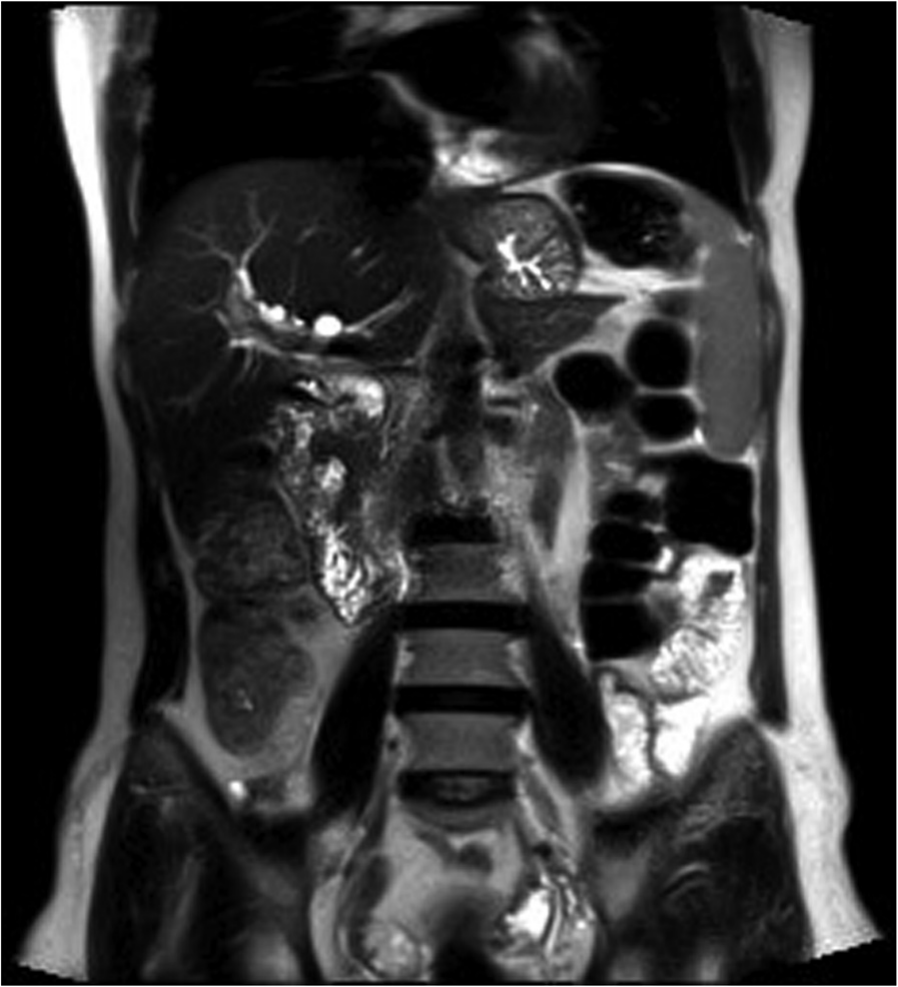
T2-weighted MRI shows the hypointense tumoral lesion in the periampullary region which has a crescent-like shape

**Fig. 4:**
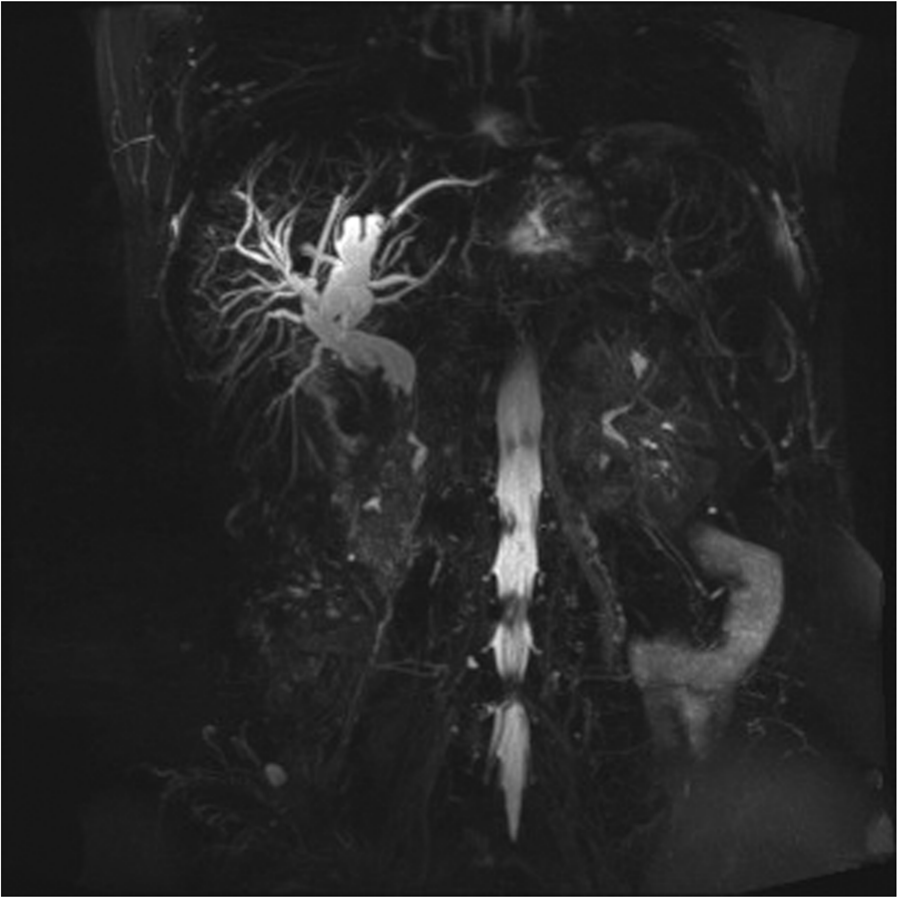
MIP image of the MRCP shows significant dilatation of the intrahepatic and proximal extrahepatic bile ducts with maximum dimension of 14 mm. Note that the tumoral lesion extends to the distal part of extrahepatic bile duct

Magnetic resonance imaging (MRI) and magnetic resonance cholangiopancreatography (MRCP) examinations demonstrated a T1 hypointense lesion with a size of 43 × 43 mm in the periampullary region occluding the distal segment of the common bile duct (Figs. 2, 3, and 4).

**Fig. 5:**
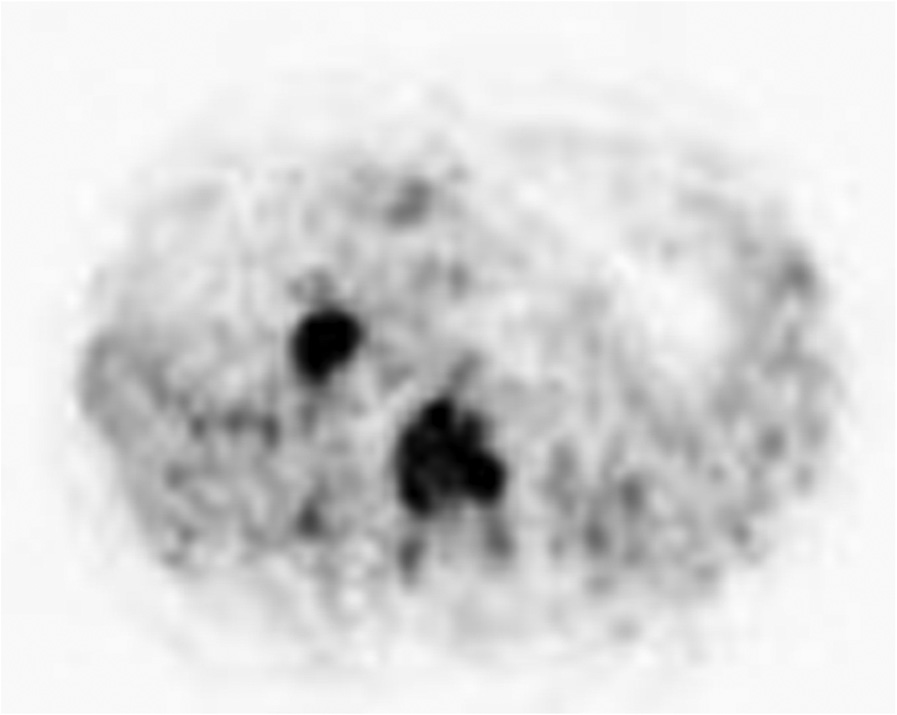
In axial PET image, FDG 18 (fluorodeoxy-glucose) uptake is seen in the periampullary region

Because of the low incidence of squamous cell carcinoma in the periampullary region, primary malignancies of other organs were also explored. Positron emission tomography (PET CT) revealed FDG (fluorodeoxy-glucose) uptake only in the periampullary region of the pancreas (Fig. 5).

The patient underwent an explorative laparotomy. Upon confirmation of neither lymphovascular invasion nor solid organ metastases, we decided to proceed with Whipple’s procedure. The postoperative course of the patient was uneventful. The patient was discharged, and adjuvant chemotherapy was recommended.

**Fig. 6:**
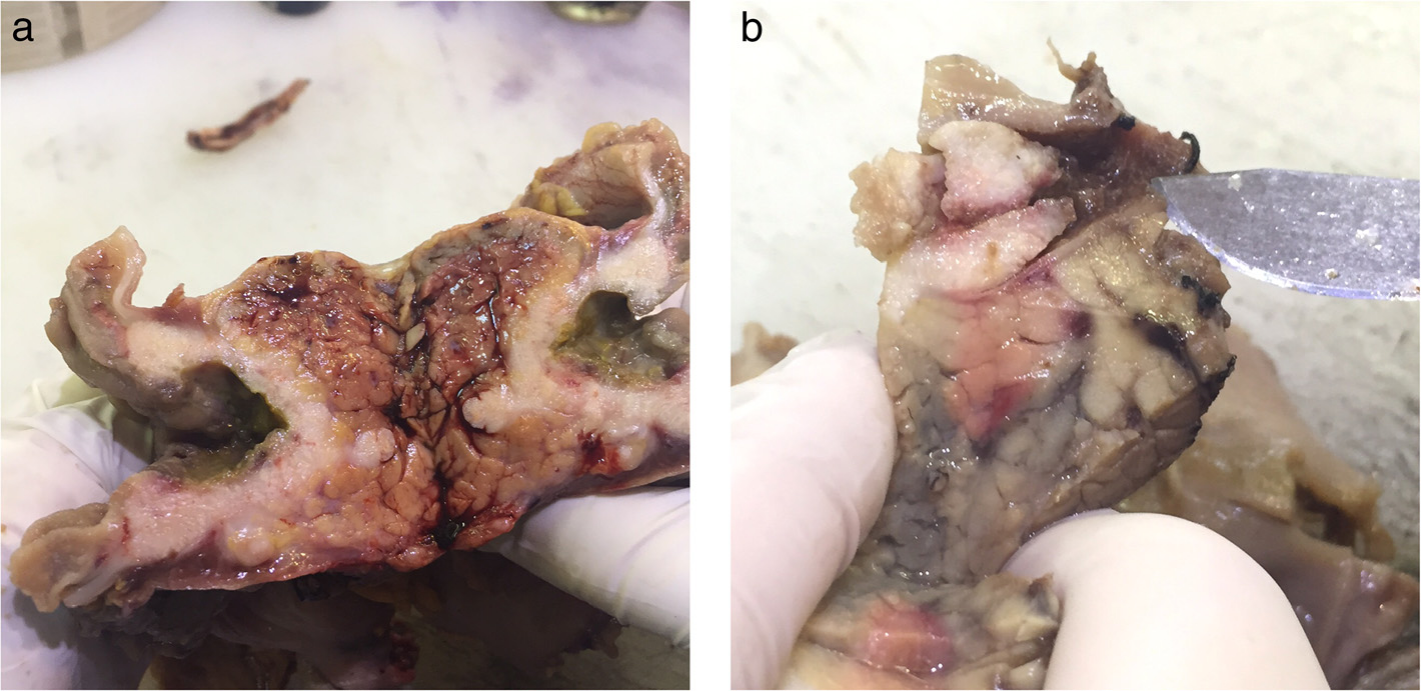
**a** Tumor location in the common bile duct. **b** Tumor invasion to the duodenum (on the *left*) and to the pancreas (on the *right*)

The histopathological examination demonstrated a moderately differentiated squamous cell carcinoma of periampullary tumor with a size of 3.7 × 3.1 × 2.1 cm, invading the duodenum and pancreas (Fig. 6).

Multiple serial sections of the tumor specimen failed to detect any adenomatous component. Although there were no signs of lymphovascular invasion, perineural invasion was present in the samples. Upon these findings, the tumor was staged as pT3N0M0.

**Fig. 7:**
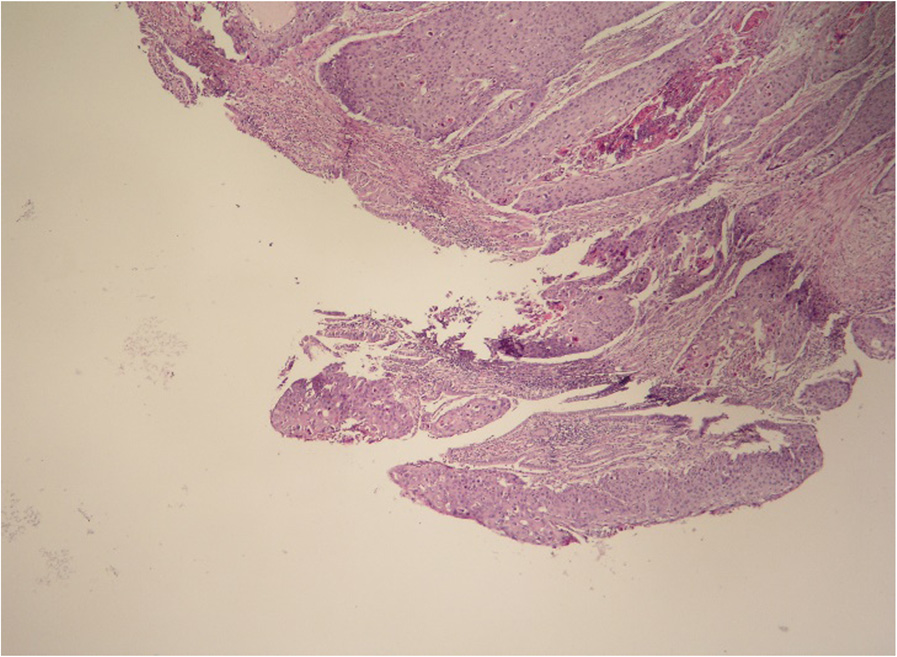
Tumor invasion to the common bile duct

**Fig. 8:**
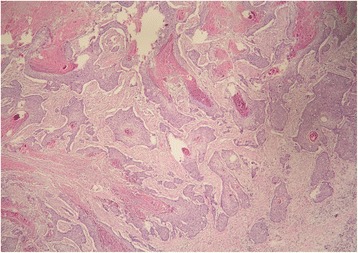
The presence of keratin pearls within the islets of atypical squamous cells

**Fig. 9:**
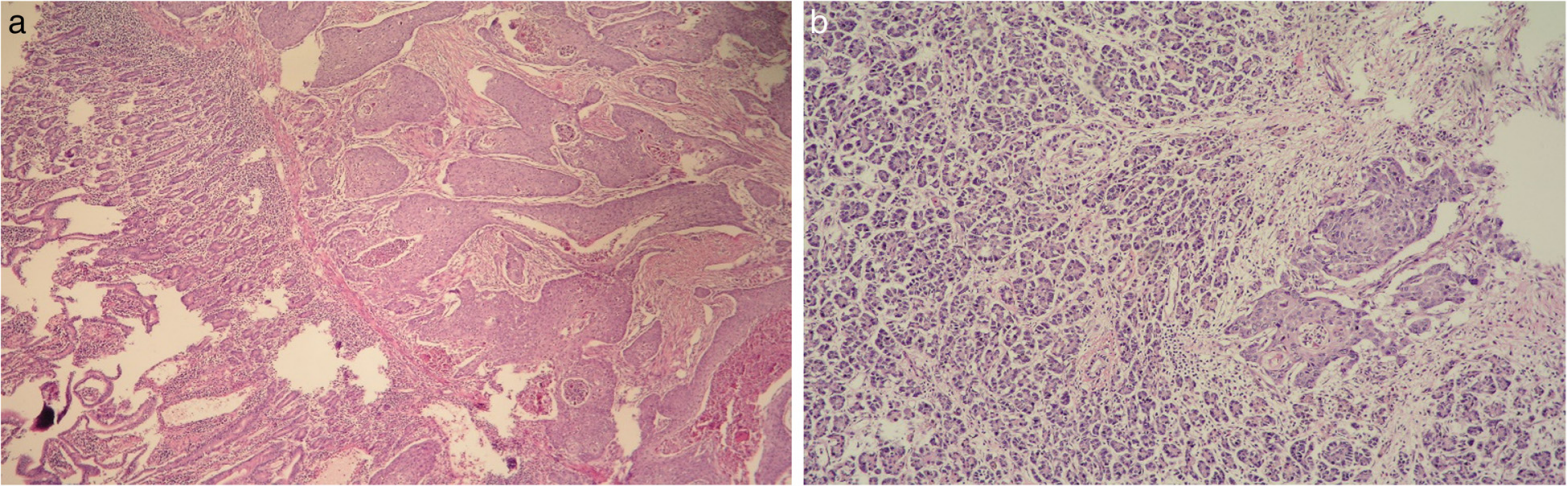
**a** Tumor invasion to the muscular layer of the duodenum and **b** to the pancreas

**Fig. 10:**
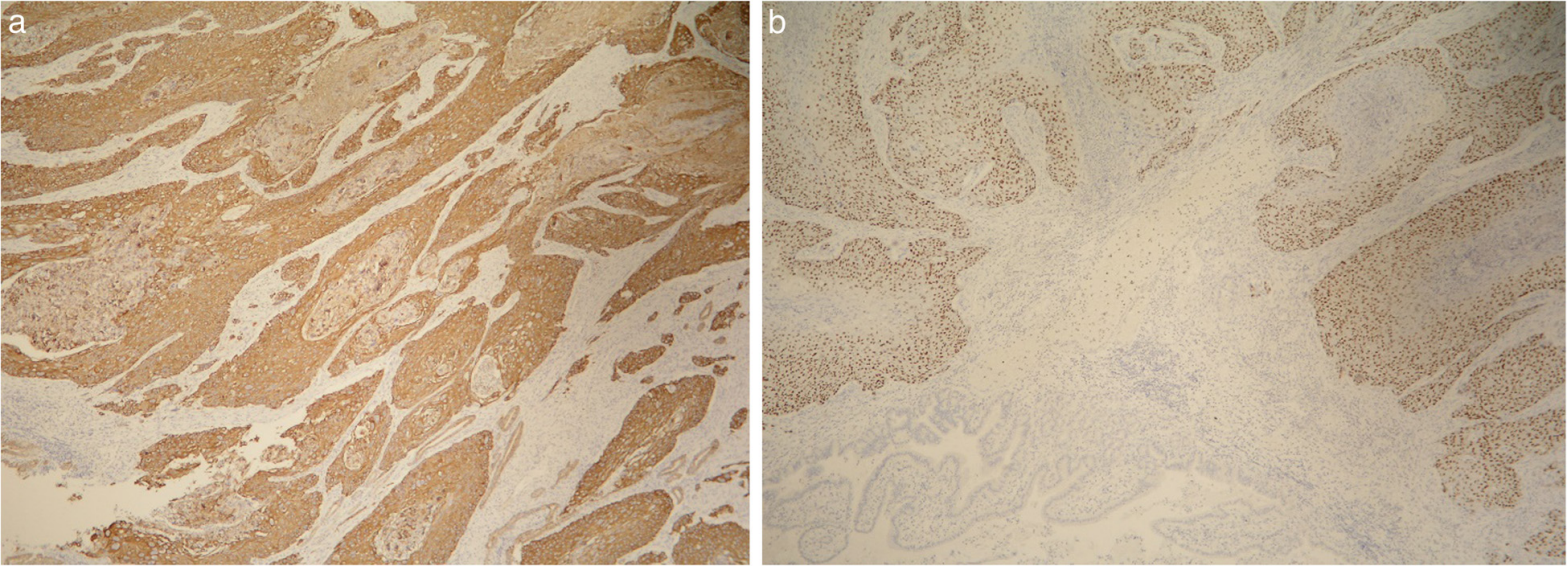
**a** Positive staining with p63 and **b** HMWCK

Immunohistochemical analysis showed that tumor cells were positive for p63 and high molecular-weight cytokeratin (HMWCK). To exclude other possible origins of primary squamous cell carcinoma, additional immunohistochemical staining analyses were performed. Tumor cells were negative for synaptophysin and chromogranin, ruling out neuroendocrine origin. Similarly, thyroid transcription factor-1 (TTF1) and CK19 were negative, excluding primary squamous cell cancer of the lung and cholangiocarcinoma. Photomicrographs of the resected specimen are shown in Figs. 7, 8, 9, and 10.

### Discussion

Distal common bile duct, ampullary, and duodenal cancers are less common than pancreatic cancer [1]. The most common histopathological type of tumor in the ampulla of Vater is adenocarcinoma. Other primary tumors that have been reported in the ampulla of Vater are squamous cell carcinoma, neuroendocrine carcinoma [10, 11], and signet cell carcinoma [12, 13]. There are only four case reports with primary squamous cell carcinoma [5–8] and one case report with co-existent primary squamous cell carcinoma and adenocarcinoma in the ampulla of Vater [9].

Because of the rarity of primary squamous cell carcinoma in the ampulla of Vater, other primary squamous cell malignancies must be excluded in all the patients. Buyukcelık et al. reported a case of squamous cell carcinoma of the larynx [14], and Sreenarasimhaiah and Hoang reported a case of esophageal squamous cell carcinoma metastasized to the ampulla of Vater [15]. Therefore, in the present case, extensive imaging studies with CT, MRI, and PET CT were performed to rule out other possible origins.

There are also few case reports of neuroendocrine carcinoma of the ampulla of Vater with squamous cell components [10, 11]. Sugawara et al. reported a case of small cell neuroendocrine carcinoma of the ampulla of Vater with foci of squamous differentiation. In their case, immunohistochemical analyses including synaptophysin, chromogranin, neuron-specific enolase (NSE), and Leu-7 were performed to identify neuroendocrine cells, and squamous cell carcinoma components were weakly positive for NSE [10]. In our case, after revealing squamous cell carcinoma by HMWCK and p63, additional staining analyses with synaptophysin and chromogranin were performed to exclude a neuroendocrine component.

The ampulla of Vater is normally devoid of squamous cells. Although the malignant transformation of ectopic squamous epithelium, the differentiation of the duodenal pluripotent stem cells [16], and squamous metaplasia secondary to chronic inflammation [17] are all among the proposed mechanisms, the exact pathogenesis of primary squamous cell carcinoma in the ampulla of Vater is still unknown.

Treatment options for periampullary tumors are surgical resection, operative or nonoperative palliation, and neoadjuvant or adjuvant therapies regardless of histopathology of tumor. Surgical resection which was also the choice of treatment in our case is the major treatment method for periampullary tumors. In another case of primary squamous cell carcinoma of the ampulla of Vater, the patient underwent curative resection without any further treatment and overall survival was 5 months after surgery was reported [5].

With limited experience of primary squamous cell carcinoma in the ampulla of Vater, long-term survival rates are not well known. On the other hand, pure squamous cell carcinomas of the biliary tract are associated with decreased survival rates compared to adenocarcinomas and adenosquamous carcinomas [18, 19]. Therefore, we suggest that primary squamous cell carcinomas of the ampulla of Vater should be considered as more aggressive than adenocarcinomas, and adjuvant chemotherapy should be recommended as another treatment option.

Different adjuvant chemotherapy regimens have been investigated for metastatic and advanced ampullary adenocarcinomas in recent years. Shoji et al. reported a retrospective study comparing 5-fluorouracil-based regimens with gemcitabine-based regimens for median progression-free survival and median overall survival time in patients with advanced ampullary adenocarcinomas [20]. Median overall survival time was found to be longer with gemcitabine-based regimens. A phase II study evaluated the efficacy of a combination regimen of capecitabine with oxaliplatin in advanced ampullary and small bowel adenocarcinomas [21]. The response rate for this regimen was lower in the ampullary adenocarcinomas compared to small bowel adenocarcinomas. This difference was suggested to be related with the heterogenous epithelium of origin and the molecular heterogeneity for ampullary tumors. In many centers, the general approach to periampullary cancers has been to use gemcitabine-based regimens for pancreatic and biliary carcinomas and fluorouracil-based regimens for duodenal and ampullary carcinomas which is also the choice of treatment in our center.

## Conclusions

Primary squamous cell carcinoma of the ampulla of Vater is a very rare histological type with an unclear pathogenesis mainly due to a limited number of cases reported. A better understanding of pathogenesis might be helpful in optimizing the treatment for this specific rare type of tumor.

## Consent

Written informed consent was obtained from the patient for publication of this case report and any accompanying images. A copy of the written consent is available for review by the Editor of this journal.

## Abbreviations

CT: computer tomography
ERCP: endoscopic retrograde cholangiopancreatography
HMWCK: high molecular-weight cytokeratin
MRCP: magnetic resonance cholangiopancreatography
MRI: magnetic resonance imaging
NSE: neuron-specific enolase
PET CT: positron emission tomography
TTF1: thyroid transcription factor-1
